# Chip Calorimetry for Single-Cell Analysis: Advances, Challenges, and Opportunities

**DOI:** 10.3390/s26072193

**Published:** 2026-04-01

**Authors:** Yara Abdelaal, Luis Guillermo Villanueva

**Affiliations:** Advanced NEMS Laboratory, École Polytechnique Fédérale de Lausanne (EPFL), Route Cantonale, 1015 Lausanne, Switzerland; guillermo.villanueva@epfl.ch

**Keywords:** isothermal titration calorimetry, differential scanning calorimetry, photothermal biosensing, thermistor, resistive temperature detector, thermocouple, thermopile, bimaterial cantilever, microresonators, MEMS

## Abstract

Heat is a crucial factor in all biological processes; therefore, measuring heat change can be a powerful tool for monitoring bioprocesses and metabolism, analyzing biomolecular interactions, and studying cells. The insights gained from thermal measurements can also aid healthcare applications, such as drug susceptibility testing and disease diagnosis. Calorimetry, the science of measuring heat, has seen many advances. However, the pressing need for miniaturization, combined with breakthroughs in micro- and nanofabrication, has led to the development of chip calorimeters and accelerated their innovation. In this comprehensive review, we discuss significant advances in chip calorimetry, including figures of merit, various applications, and key challenges. The review offers an overview of the current state of the art, highlighting prospects and opportunities.

## 1. Introduction

Calorimetry plays a vital role in understanding chemical and biological processes. On a fundamental level, it can be used to evaluate thermodynamic parameters, analyze binding and reaction equilibria, characterize phase and conformational transitions, understand solvent interactions, characterize enzymatic reactions, and measure cell metabolism [1]. From there, these capabilities can be applied to a plethora of different fields, including environmental studies, food industry applications, and healthcare [2].

Building on the latter, medicinal compounds, such as drugs and vaccines, can be developed based on the quantitative assessment of the thermodynamic parameters governing different intermolecular phenomena [3,4]. Moreover, direct measurement of bacterial metabolic activity enables real-time detection of inhibitory effects and, consequently, bacterial susceptibility to antibiotics [5]. Additionally, calorimetry is proven as an efficient tool for diagnosis and prognosis, as it can be used in the detection, monitoring, and treatment testing of various cancer forms [6,7,8,9,10,11], as well as for differentiating subtypes and predicting survival [12]. Furthermore, it can contribute to reducing the time necessary for bacterial detection [13] and accurate infection diagnosis and aid efficient treatment [14].

Such advances have only been enabled by continuous innovation in calorimetric tools. The two most widely used calorimetric techniques are Isothermal Titration Calorimetry (ITC) and Differential Scanning Calorimetry (DSC), both capable of directly measuring the enthalpy change in a sample [3]. For both techniques, various systems have been developed, and numerous enhancements have been implemented over the years. This resulted in improved reliability and repeatability, enabling applications such as measuring protein binding energetics [15] and analyzing the plasma proteome [16], among numerous others. We dedicate the subsequent subsections to further elucidating the working principles of ITC and DSC.

### 1.1. Isothermal Titration Calorimetry

ITC is regarded as the gold standard for investigating molecular interactions in solutions, owing to its direct measurement of heat exchange under isothermal conditions [3]. The system comprises two chambers: a sample chamber and a reference chamber containing a buffer solution, both housed in an adiabatic shield. The technique relies on titrating a binding parameter (the titrant) into a solution of its interaction partner (the titrand) within the sample chamber. The titrant is incrementally injected into the sample chamber at predefined time intervals, and the heat absorbed or released upon each incremental addition is measured relative to the reference vessel. The measurement is electrical, with the principles thoroughly explained in Section 3. The amount of power (in W) required to restore thermal equilibrium, corresponding to the enthalpy change, is recorded.

**Figure 1:**
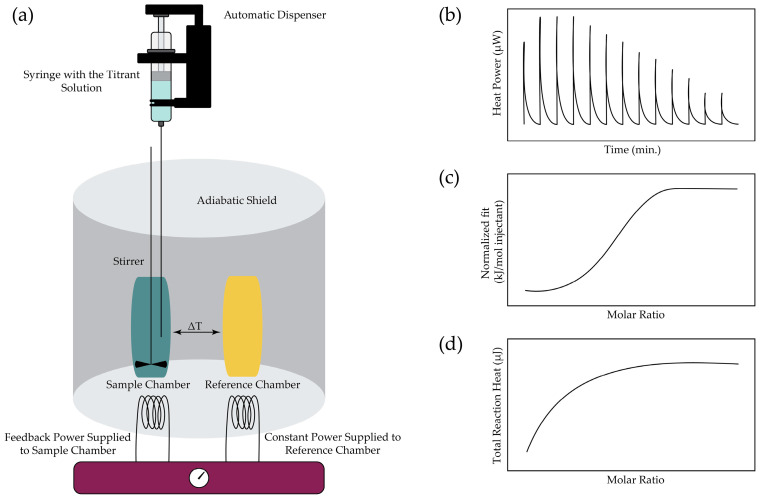
Principle of Isothermal Titration Calorimetry [18,19]: (**a**) Schematic of the calorimeter set-up; (**b**) The extracted raw data; (**c**) The differential plot of the data; and (**d**) The integral plot of the data.

The obtained data is typically represented in two ways: the differential and the integral plots [17]. In the differential plot, the total heat accumulated up to a given injection is normalized to the ligand concentration at that point and plotted either against that concentration or against the concentration ratio of total ligand to total receptor. This yields the characteristic sigmoidal titration curve used to derive the total calorimetric heat change per mole. In contrast, the integral plot presents the cumulative heat as a function of the total ligand concentration or the ratio of total ligand to total receptor, yielding a hyperbolic saturation curve from which equivalent thermodynamic parameters can be extracted, as shown in Figure 1.

### 1.2. Differential Scanning Calorimetry

DSC is a technique employed to determine a specimen’s heat capacity as a function of temperature, and to identify thermal transitions such as melting, crystallization, and denaturation. A standard DSC system consists of twin chambers: one designated for the sample and the other for the reference. The tool is equipped with a temperature sensor and connected to a heating module. In contrast to ITC, which measures heat changes during titration at a constant temperature, DSC operates by scanning the temperature. The system operates by either heating or cooling the cells at a constant rate and monitoring the temperature difference between the two chambers, which reflects the changes in the sample’s heat capacity or other thermodynamic events.

There are several types of DSCs, including: the Power-Compensated DSC, the Heat Flux DSC, and the Temperature Modulated DSC (TMDSC) [16]. The Power-Compensated DSC directly measures the electrical power (W) required to maintain the sample and reference at identical temperatures [20]. The Heat Flux DSC records the temperature difference between the chambers to convert it into a heat flow difference based on the known thermal resistance [21]. The TMDSC, alternatively, introduces a low-frequency modulation to the thermal heating profile [22], offering optimization through the adjustment of the average scanning rate, modulation period, and amplitude [16]. The heat Flux and Power-Compensated DSC are the most commonly employed configurations, yielding identical data despite their differing methods of operation [23].

**Figure 2:**
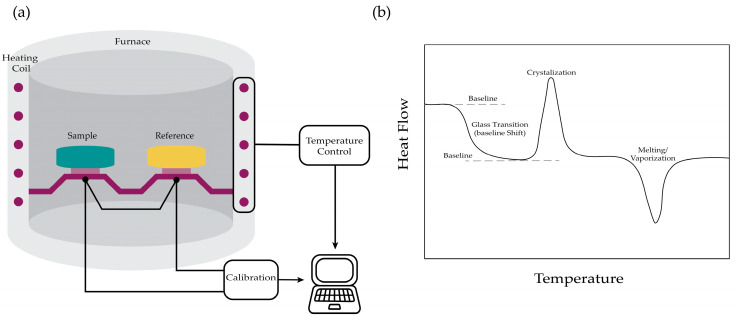
Principle of Differential Scanning Calorimetry [19]: (**a**) Schematic of the calorimeter set-up; (**b**) The extracted raw data.

DSC produces plots of heat flow as a function of temperature, in which thermal events manifest as deviations from the stable baseline. In Heat Flux DSC, the signal is derived from the temperature difference between sample and reference, resulting in a smoother, more stable baseline that is ideal for detecting subtle changes. Conversely, Power-Compensated DSC measures the power required to maintain equal temperatures, enabling faster scan rates and sharper peak resolution at the expense of baseline stability. The onset temperature, which indicates the initiation of the transition, and the peak temperature, which represents the maximum energy change, serve as key markers of transition points. The area under the curve corresponds to the enthalpy change. Additionally, baseline shifts reflect differences in heat capacities, aiding in the identification of glass transitions or phase behaviors, see Figure 2.

### 1.3. The Need for Miniaturization

Historically, the two calorimetric techniques (ITC and DSC) were first implemented in various bench-top systems, bringing numerous benefits to biomedical and pharmaceutical sensing, among many other fields. The continuously advancing ITCs and DSCs, capable of converting heat changes from different interactions into electrical signals, have offered significant flexibility by accommodating a wide variety of sample types, regardless of opacity, porosity, or phase. Moreover, they offer notable convenience through their noninvasive and label-free operations. However, this advantage can be a double-edged sword, as the resulting signal may include contributions from nonspecific binding and non-isolated thermal events from the surroundings [24]. This is not the only challenge facing these bench-top systems; they also exhibit several limitations. These limitations can be summarized as: (1) slow measurements, (2) requirement of substantial sample volumes, (3) high cost, and, most notably, (4) the limited thermal insulation resulting in a limited resolution.

Miniaturization has emerged as a robust strategy to overcome these constraints, particularly considering the rapid advancements in micro- and nanofabrication and the integration of microfluidics that enable micro-Total Analysis Systems (µTAS). The transition from bench-top calorimeters to chip calorimeters has resulted in faster measurements by reducing measurement time constants (from an average of 30 min to seconds and milliseconds) and facilitating array measurements. Moreover, this shift has contributed to a reduction in sample volume requirements to nanoliters (nL) and picoliters (pL) [25]. Additionally, mass production has lowered fabrication costs and facilitated the development of disposable calorimetric sensors, thereby minimizing cross-contamination [24].

In the context of applying calorimetry for cell analysis, two pivotal points must be emphasized: first, bulk cell measurements are inherently limited. In addition to the requirement for sufficient spatial and temporal resolution to identify potential local hotspots, it has been conclusively demonstrated that population-level averaging is not representative of single-cell behavior. This approach masks extreme behaviors and overlooks rare yet biologically significant cells [26]. Consequently, the need to enhance the resolution of chip calorimeters for single-cell analysis has become central, prompting substantial advances in the field. To illustrate the required resolution range, Table 1 summarizes the reported heat power values for single living cells and organisms from the literature.

The second pivotal point is the essential need for microfluidic integration, as measuring biological entities must be conducted in their native environment, which is primarily aqueous. Furthermore, it is well-established that cellular function is inherently influenced by dynamic cell–cell communication mediated through proteins, nucleic acids, extracellular vesicles, and small molecules [30]. By enabling confinement and pairing through microfluidic platforms with spatial and temporal precision, a framework is provided to understand how signaling exchanges influence cellular behavior at the single-cell level. Including such perspectives is crucial when interpreting single-cell measurements, such as thermal signatures, within a biologically relevant context.

This review paper aims to trace the major innovations in chip calorimetry intended for biological applications and explore the possibility of achieving single-cell resolution. The analysis begins with a detailed review of reported technologies, categorized by their thermometry principles: optical, electrical, or mechanical. It should be noted that this classification does not encompass entire chip systems, as each chip calorimeter is typically a hybrid assembly of components designed to manage measurement, signal processing, insulation, and sample handling. Our categorization, however, focuses solely on thermometry. We highlight the achieved figures of merit, current applications, and subsequently the major challenges and future opportunities.

Although prior reviews on chip calorimeters have been published, each addressing specific aspects of the topic, the present work offers an integrative and distinct perspective. The contribution of A.W van Herwaarden [31] introduced a range of early chip calorimeters based on electrical thermometry principles. The subsequent article by Lerchner et al. [32] likewise exclusively highlighted chip calorimeters employing electrical sensing, emphasizing potential optimization routes to improve signal resolution and reduce sample volume, thereby enabling the analysis of enzymatically catalyzed reactions and microbial growth. The review by Shifeng Yu et al. [16] adopted a narrower scope, centering differential scanning calorimetry, where they have discussed commercially available DSC tools along with developed chip DSCs.

Some reviews have framed the topic in a broader context, addressing metabolism monitoring through cell temperature measurements [33] or reviewing microcalorimetry advancements for bioanalysis and energy balance monitoring [34]. Other works [24,35,36,37] have opted for a more narrowly focused exploration of chip calorimeters. These latter studies provided elaborate categorizations based on measurement invasiveness [36], thermal insulation approaches [24,35,36,37], sample handling techniques [24,35,36,37], reaction chamber design [35,36], mode of operation [24], sensing configuration [36], and materials employed [35].

While we fully recognize the value of such thorough classification, we choose to adopt a classification centered on the heat-sensing principle. This approach enables a more directed and chronological structure for the overview of each of the three sensor types mentioned above, in addition to facilitating clearer comparison and benchmarking within each category. By adopting this categorization, a thorough analysis of different systems becomes possible through evaluating their design, operation, and their suitability for achieving single-cell calorimetry. Furthermore, this review extends previous works on chip calorimeters that place greater emphasis on electrical sensing systems, by particularly highlighting mechanical chip calorimeters, which we identify as highly promising and competitive candidates for achieving single-cell resolution.

## 2. Optical Chip Calorimeters

Optical techniques, when employed for calorimetry, can provide reliable, non-invasive tools with high spatial and temporal resolution. The systems exhibit considerable variability in their operational modes. Consequently, the selection of the technology depends on the intended application, which dictates the resolution specifications and limitation tolerances. This section provides an overview of the technologies reported in the literature, along with a discussion of their diverse applications and challenges.

### 2.1. Infrared Thermography

Infrared (IR) thermography relies on monitoring the radiation emitted by a heated body and can measure temperatures in the range of 50 to 6000 K. The measurement system consists of the target analyte, the medium through which the radiation is transmitted, and the detection apparatus. This apparatus, in turn, comprises the detector and a control and analysis system [38]. Early work has employed an IR thermography system for the clinical diagnosis of rheumatoid and vascular conditions [39].

This technique—in its bulk form—was further applied to miniaturized systems, including for non-contact thermal monitoring and control of a Polymerase Chain Reaction (PCR) microfluidic chip [38], as well as for studying thermal profiles in microchannel reactors [40] and chip microcalorimeters [41]. It was also shown to be scalable using a single-crystal silicon diode [42]. Nonetheless, the wavelength range of this technology has constrained its applications on the chip level. Since chip calorimetry is often conducted in aqueous environments, measurement resolution is inherently limited by both absorbance and diffraction effects [43].

### 2.2. Refractive Index Calorimetry

Measurement of refractive index change can also be employed to detect thermal variations, as it is a measurable parameter that monotonically varies with temperature. Several systems have been developed to utilize this phenomenon to detect local heat alterations [44,45,46,47,48,49].

### 2.3. Liquid Expansion Calorimetry

Similar to the principle of a household mercury-in-glass thermometer, a type of microcalorimeter was developed that relies on the thermal expansion of a confined sample liquid in a narrow capillary [50,51,52]. The 1–2 µL sample is introduced as a droplet into a sealed, humidity-controlled capillary to minimize evaporation-related errors, and the analyte’s meniscus is monitored using a Michelson interferometer, enabling real-time detection of heat changes in the µJ range.

### 2.4. Liquid Crystal Calorimetry

Liquid Crystals (LCs) are an intermediate state of matter between crystalline solids and isotropic liquids, exhibiting long-range order while maintaining fluidity. This exceptional characteristic arises from the uniform directional orientation of their molecules within specific regions known as “domains”, with each domain displaying distinct molecular alignment [43,53]. This review is primarily concerned with Thermotropic liquid crystals, in which, as indicated by their nomenclature, temperature is the sole thermodynamic parameter governing their phase. These liquid crystals comprise a single molecular species [53] and can be classified, based on molecular orientation, into nematic and cholesteric LCs.

In nematic LCs, anisometric molecules (rod- or disc-shaped) lack positional order but align along a common axis: the director *n*. Temperature variations can induce a nematic-isotropic phase transition, leading to changes in optical birefringence or light transmission. The nematic phase scatters light, the isotropic phase is transparent and appears as dark spots under cross-polarizers. The boundary between the birefringent and dark spots defines an isotherm corresponding to the transition temperature, known as the clearing point T_pt_ [54]. The use of nematic LCs in calorimetry is limited by their ability to detect only a single isotherm. This constraint can be overcome by placing the sample in a micro-thermostat and tracking isotherm displacement during controlled temperature changes, enabling thermal mapping [54]. Alternatively, nematic LCs can be used for binary detection of biological events, such as DNA hybridization [55].

In comparison to nematic LCs, cholesteric LCs possess an additional one-dimensional positional order [53], with molecules arranged in layers whose director gradually rotates from one layer to the next, forming a helical structure. The pitch—defined as the distance over which the director makes a full 360° rotation—determines the selective reflection of specific wavelengths based on the angle of view. Temperature variations alter the pitch, leading to corresponding shifts in the reflected color. Consequently, the temperature can then be inferred from the hue or the wavelength of maximum reflectivity [43], which is why these crystals are also termed thermochromic LCs.

Thermochromic LCs have been utilized in miniaturized systems for spatially resolved, real-time temperature monitoring during chip-based PCR cycling [56]. They were additionally employed for measuring the enthalpy change in reactions using a segmented droplet microfluidic chip [57].

### 2.5. Fluorescence Calorimetry

Fluorescence refers to the emission of light by a molecule after the absorption of electromagnetic energy. This phenomenon occurs when an excited electron, energized by the absorbed radiation, returns to its ground state [58]. Numerous materials exhibit highly temperature-dependent fluorescence properties, thereby promoting their use as remote and highly sensitive nanothermometers [59]. A variety of organic and inorganic thermosensitive fluorescent materials have been developed. Their thermal response can vary, but it remains measurable via optical indicators, including emission intensity, lifetime, peak position, Electron Spin Resonance (ESR), and Optically Detected Magnetic Resonance (ODMR), among others [59].

Temperature-responsive fluorescence materials have been mainly employed for contactless intracellular and in vivo measurements, namely, the detection of the thermally induced spectral shift of a Quantum Dot (QD). Nitrogen vacancy (NV) centers in diamond have been similarly employed, relying on the detection of ESR or ODMR [60,61]. An alternative approach would be to draw thermal measurements upon the material’s photoluminescence lifetime, as previously reported for intracellular thermometry using polymeric fluorescent [62] or Carbon Dot [63] nanocalorimeters. Additionally, fluorescence can be employed in parallel to a chip DSC system, offering dual-mode calorimetry measurements [64].

### 2.6. Electroluminescence Calorimetry

In a related approach, electroluminescence was also utilized for chip calorimetry in what has been termed an Optical Wireless-Integrated Circuit (OWiC) [65,66]. The device consists of an array of Photovoltaic cells (PVs) that power a connected AlGaAs/GaAs Light-Emitting Diode (LED) when illuminated. As the LED intensity is proportional to the temperature, the device can be employed for direct thermal sensing [66].

### 2.7. Photothermal Biosensing

Another significant technology is the use of nanomaterial-mediated photothermal effects in biosensing. In these systems, the change in heat is not the target quantity for measurement; rather, it serves as an intermediate step in transduction. The idea relies on Localized Surface Plasmon Resonance (LSPR), referring to the resonant oscillation of conduction electrons at the surface of metallic nanoparticles. Upon shining light with a specific wavelength on nanospheres, nanorods, or nanocages, localized heating is induced. In these photothermal biosensing platforms, the nanoparticles serve as probes, and the induced heat is either optically detected or converted into a visual readout, but in either case, it quantitatively correlates with the target metric.

This approach was implemented in combination with a Lateral Flow Assay (LFA)—a paper-based diagnostic device [67]. Gold nanocages served as labels for sandwich or competitive immunoassays, with heat measured via an IR camera, offering a rapid, sensitive, and accurate diagnostic platform. In a similar strategy, gold Nanoparticles (NPs) were employed in a quantitative genetic detection platform using a standard thermometer for heat measurement [68]. Another device utilized Fe_3_O_4_ NPs for immunosensing, and the resulting heat signal was converted into a visual readout, enabling an instrument-free Lab-on-chip (LOC) analytical application [69].

### 2.8. Design and Performance Considerations

**Figure 3:**
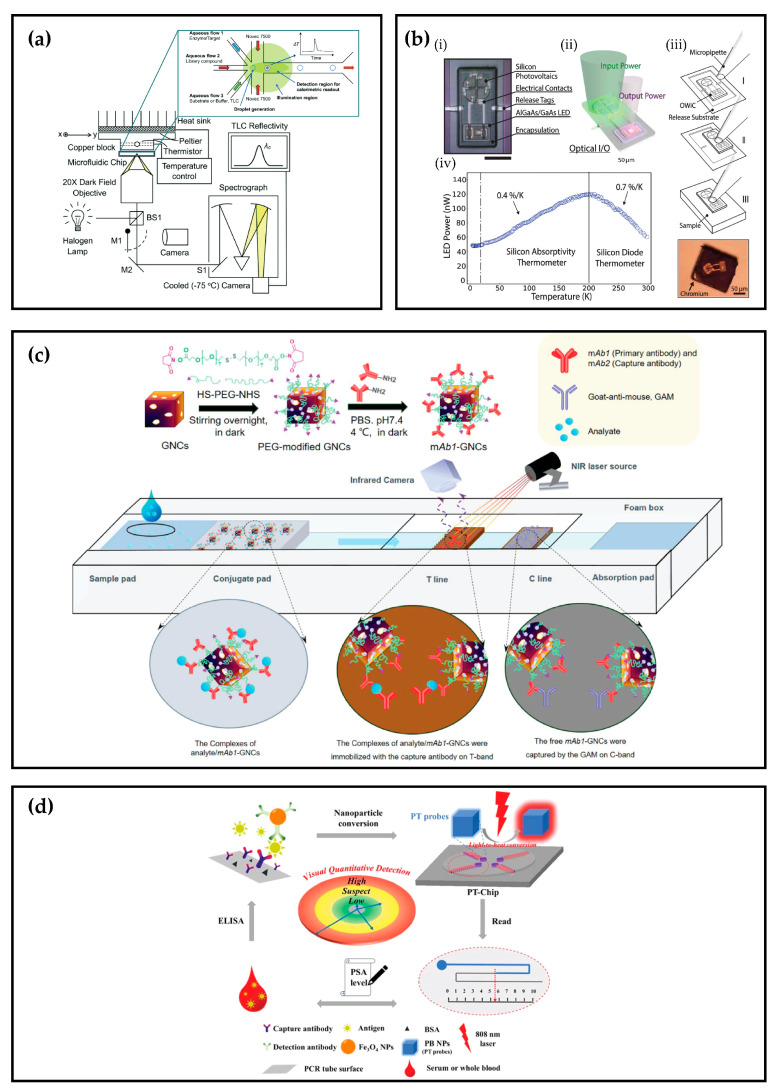
Optical Chip Calorimeters and their different working principles: (**a**) Experimental Set-up to track the temperature shift of picodroplets undergoing chemical reactions employing thermochromic LCs [57]; (**b**) OWiC thermometer where (i) is a microscopic image of the device, (ii) shows the optical I/O of the OWiC, (iii) is a schematic of the pick and place method through steps I to III and the device placed directly on a chromium chip, and (iv) illustrates the OWiC behavior from 4 k to 300 K [66]; (**c**) Schematics of the calorimetric LFA employing gold nanocages [67]; (**d**) Working principle of the nanomaterial-mediated photothermal bar-chart microfluidic for visual quantitative biosensing [69].

Figure 3 illustrates various chip calorimeter devices that employ different optical technologies.

Despite the wide variety of optical methods, these approaches are not without limitations. Many lack versatility, with their operation confined to specific wavelengths or temperature ranges. Furthermore, optical calorimetry is generally resistant to miniaturization, as most implementations continue to rely on bulky and expensive analytical instruments that cannot be integrated into a stand-alone chip calorimeter. An additional challenge lies in the need to prepare specialized probes—such as nanoparticles—for measurements.

## 3. Electrical Chip Calorimeters

Among the continuously evolving chip calorimeters, the electrical calorimeters are the most widely developed and utilized. Their operational principles are relatively simple and well-suited to chip miniaturization, with efforts primarily focused on optimizing signal resolution and reducing sample volumes. Numerous devices were based on scaling ITC [8,70,71] and DSC [72,73,74,75,76,77,78,79,80] technologies for chips.

Based on their working principle, electrical calorimeters can be divided into two main categories: Resistance-Based and Thermocouple-Based. Resistance-based calorimeters can be subdivided into three types: thermistors, Resistive Temperature Detectors (RTDs), and PN junction diodes. In this section, we discuss the various electrical chip calorimeter technologies, their applications, benchmark performance values, and associated challenges.

### 3.1. Resistance-Based Chip Calorimeters

#### 3.1.1. Thermistor-Based Chip Calorimeters

Thermistors are devices that use materials (typically doped semiconductors or oxides) whose resistance varies non-linearly with temperature. This variation is expressed through the Temperature Coefficient of Resistance (TCR). They offer high sensitivity and precise absolute-temperature measurement. However, the devices must be electrically powered during operation, which results in joule heating and affects their output. This effect must hence be accounted for during the calibration of the device to ensure the integrity of the measurement [81].

Some of the materials used in thermistor chip calorimeters are doped polysilicon [82,83], amorphous Si [84], and vanadium oxide (VOx) [77,79,85,86,87,88,89,90,91,92,93]. The TCR of VOx can be varied through the control of oxygen content during the material sputtering [87], but controlling its oxidation state and electrical properties is rather tricky. This is not the only limitation for the use of VOx, as the material is toxic [94].

#### 3.1.2. RTD-Based Chip Calorimeters

RTDs are typically made of metal films whose resistance also varies with temperature in an approximately linear fashion. Compared to thermistors, RTDs offer a wider temperature range of operation and measurement, higher stability, better reproducibility, and simpler fabrication, in addition to linearity [81].

Among the metal thin films employed as RTDs are Ni [72,95,96], Pt [97,98,99], Al [100,101], Ti [102], Ag [103], and Niobium nitride (NbN_x_) [104,105]. The TCR value of a metal thin film is a function of the ratio between the film’s thickness and the electron’s free path in metals. The coefficient’s value, hence, depends on the fabrication process [37].

Common mode, background drift, and noise can affect the measurement of both thermistors and RTDs equally. To suppress the influence of these factors, a balanced Wheatstone bridge can be employed [87,95].

#### 3.1.3. PN Junction-Based Chip Calorimeters

PN junction diodes can similarly be used for chip calorimetry, owing to the temperature dependence of the diode’s resistance [106]. The device offers a high TCR of 1.4%/K and is well-suited for downsizing, capable of resolving temperature and heat changes of 1 mK and 75 nW, respectively.

**Figure 4:**
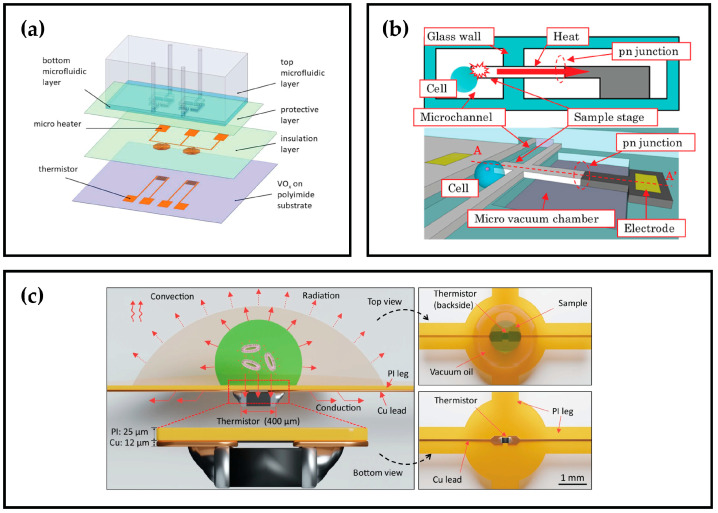
Examples of Resistor-based Chip Calorimeters: (**a**) A MEMS DSC employing Vox thermistors for the study of protein stability [93]; (**b**) Schematic of the PN junction thermal microsensor illustrating the cross-sectional view (on the top), as well as the overall view (on the bottom), where the white part is the n-type area and the gray is the p-type area [106]; (**c**) A chip calorimeter where a commercial thermistor is integrated on a flexible printed circuit board, and the sample is covered with oil to avoid evaporation [107].

Figure 4 illustrates examples of chip calorimeters that rely on thermistors, RTDs, or PN Junctions. Table 2 also summarizes some of the devices reported in the literature, categorizing them by their type, materials, and TCR.

### 3.2. Thermocouple and Thermopile-Based Chip-Calorimeters

Thermocouples—and their series connection to form thermopiles—are devices whose working principle is based on the Seebeck effect. The Seebeck effect is a thermoelectric phenomenon in which thermal energy is converted into electric energy. When a temperature gradient is created in a conductor or semiconductor material, carriers will diffuse within the material, causing electrons to move from the higher-temperature side to the lower-temperature side, thereby inducing an electric potential difference (∆V) [108,109]. The induced voltage (∆V) is proportional to the temperature gradient (∆T) and expressed as:

(1) ∆V=S∆T,

where S is the Seebeck coefficient, which similarly varies with temperature. This variation needs to be accounted for in cases of significant temperature variations. Table 3 reports the Seebeck coefficients of commonly used materials relative to Pt whose coefficient is −5 µV/K.

**Figure 5:**
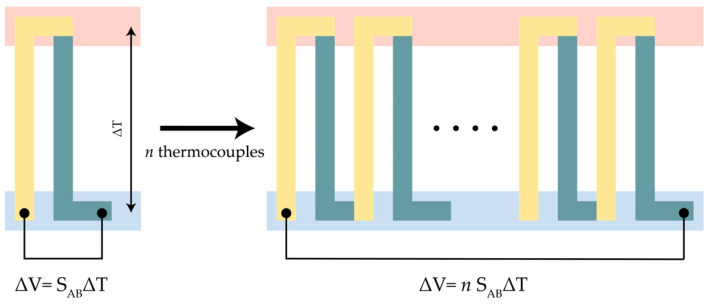
Thermoelectric effect for a thermocouple and a thermopile (reproduced from [115]).

A thermocouple typically consists of two dissimilar materials connected at a junction. The relative Seebeck coefficient $S_{AB}$ of the thermocouple is then defined as the difference between the individual Seebeck coefficients of the materials used [113], and is dependent on the material’s composition and the working temperature [114]. For the small temperature ranges considered by chip calorimetry, the relative Seebeck coefficient can thus be regarded as constant. In chip calorimeters, such devices are made by depositing and connecting thin-film materials, commonly known as Thin Film Thermocouples (TFTCs). A thermopile is created by connecting multiple thermocouples in series, and the induced voltage equals the sum of the voltages from each thermocouple, as shown in Figure 5. The induced voltage is expressed as:

(2) $$∆V=nS_{AB}∆T,$$

where n is the total number of thermocouples, $S_{AB}$ is the thermocouple relative Seebeck coefficient as illustrated in Figure 5.

**Figure 6:**
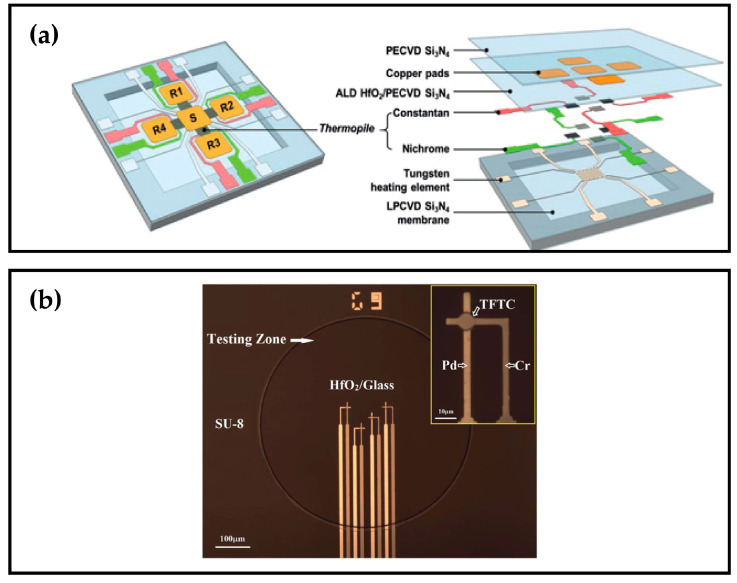
Examples of thin film thermocouple devices: (**a**) A schematic of the overall chip calorimeter, including the sample (S) and four reference pads (R_1_ to R_4_), and the sequence of layers within the sensor [115]; (**b**) A microscopic photograph of a TFTC device with a 600 µm testing zone and 4 Pd/Cr micro TFTCs, with the inset presenting a single thermocouple for details [156].

Numerous material pairs have been employed as thermocouples, namely Ti/Bi [25], Bi/Pt [29], Cr/Ni [116], Au/Si [117], Au/Pd [118], Cr/Cu [119,120], Bi/Ti [121,122], Au/Ni [123,124,125,126], Al/Si [114,127,128,129,130,131,132,133,134], Bi/Sb [5,8,70,71,74,135,136,137,138,139,140,141,142,143,144,145,146,147,148,149,150,151,152,153,154], Constantan/Nichrome [115,155], Cr/Pd and Cr/Pt [156]. Si can additionally serve as a thermocouple material through variations in doping and crystallinity [113,157,158,159,160]. Furthermore, to circumvent the need for a two-layer deposition, thermocouple pairs can be fabricated using a single metal layer (Ni, Pd, W, or Pt), and the differentiation of the thermocouple pair is achieved through width variations in the design [161]. Some of the TFTCs devices are shown in Figure 6.

Although thin-film thermopiles are used in most chip calorimeters, they are not the only design approach. Several works presented thermocouple probes designed for insertion into the target sample, employing various metals and alloy pairs such as Au/Pt [162], Pt/W [163,164], and Constantan/Nichrome [165]. Another chip calorimeter utilized aqueous solutions as the thermocouple pair [166]. Table 4 lists some thermocouple devices reported in the literature.

### 3.3. Design and Performance Considerations

Thermocouples, in contrast to thermistors and RTDs, do not require supplementary power; therefore, no additional heat is introduced into the system. However, the fabrication of thermocouples is more intricate than that of resistive-based calorimeters, as it typically involves a two-layer deposition coupled with additional lithography steps. Although the series connection into thermopiles can significantly increase the sensor’s response, it also leads to an increase in its thermal conductance, consequently decreasing the temperature change and the calorimeter’s output voltage. Additionally, the integration of many thermocouples also dictates a larger chip calorimeter footprint, thus limiting its scaling. One way to circumvent this limitation is to construct 3D stacking of the thermocouple thin films through the use of insulating thin films [168].

TFTCs also face additional challenges related to minimization and scaling effects as well as measurement circuitry [169]. A previous study has shown that the absolute Seebeck coefficient of metallic thin films are smaller than that of their bulk counterparts, resulting in reduced thermocouple responsivity compared to theoretical expectations [170]. Further reduction in the thermocouple’s metallic stripe width to submicron and nanometer scales can cause an additional decrease in the sensitivity; however, the optimal design depends on the targeted resolution as devices with responsivity as small as 1–2 µV/K can still achieve 20–50 mK thermal resolution. Such specs, however, will dictate the use of appropriate measuring circuitry capable of detecting DC voltages in the sub-microvolt range.

It should be noted that our analysis does not include electrical chip calorimeters specifically designed for probing quantum phenomena. Using either resistive thermometry in metals or superconductors [171], or more advanced Normal Metal-Insulator-Superconductor (NIS) junction-based approaches [172,173], such devices have demonstrated exceptional resolution, reaching atto- to zeptowatt heat power at millikelvin temperatures. While these systems provide benchmarks for the ultimate detection limits achievable in chip calorimetry, a detailed analysis lies beyond the scope of this review, which focuses on calorimetric platforms for biological applications.

## 4. Mechanical Chip Calorimeters

Mechanical chip calorimeters have been gaining significant importance, especially for biological applications. Their straightforward operational principles have facilitated innovation and optimization. Furthermore, while other chip calorimeters encounter challenges related to insulation—as elaborated in the subsequent section—mechanical chip calorimeters provide insulation through their inherently suspended architecture and, in some cases, their operation in vacuum. These factors have enabled them to achieve high thermal resolution and compete effectively with their widely used electronic counterparts.

Typically, mechanical chip calorimeters are operated in either static or dynamic modes. Static mechanical chip calorimeters rely on the static bending of a suspended structure. In contrast, in dynamic chip calorimeters, the mechanical structure is actuated, and measurements are performed by monitoring the resonator frequency. This section examines the various mechanical chip calorimeter technologies, their performance metrics, and their applications.

### 4.1. Static Mechanical Chip Calorimeters

**Figure 7:**
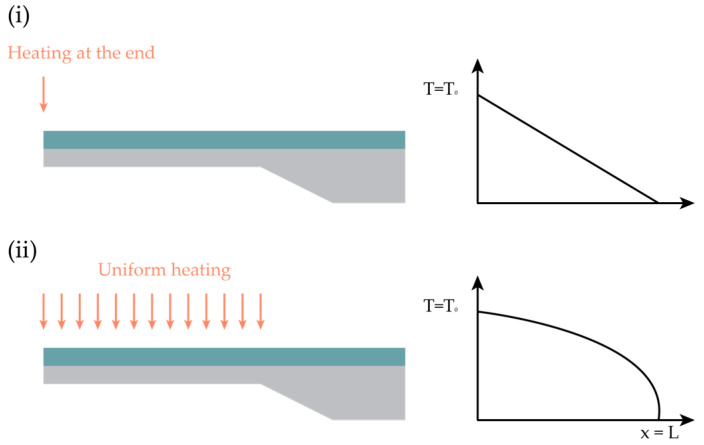
The BMC arrangement and temperature profiles along its length L for the two situations: (**i**) The heat is absorbed only at its end; (**ii**) The heat is uniformly absorbed along its length [176].

Bimaterial Cantilevers (BMCs) (Figure 7) have been widely utilized in chemical and biosensing. When employed for calorimetry, the sensing relies on the cantilever’s static bending due to the differential thermal expansion of the materials upon absorption of heat [174,175].

Early models have been formulated for the analysis of a rectangular silicon nitride/Aluminum BMC [176], where the cantilever’s fixing point is assumed to be a perfect heat sink. Two cases can be considered:

- The heat is absorbed only at the end of the cantilever, resulting in a linear temperature profile;
- The heat is uniformly absorbed along the cantilever’s length, resulting in a parabolic temperature profile.

The responsivity—defined as the deflection at the tip of the cantilever z(0) per unit of thermal power P—can be expressed for both cases by the following proportionality:

(3) $$\frac{z(0)}{P}=C_{0}\left(\gamma_{1}-\gamma_{2}\right)\frac{{l^{3}(t}_{1}+t_{2})}{w\;t_{2}^{2}K\;(\lambda_{1}t_{1}+\lambda_{2}t_{2})},$$

where $C_{0}$ is a constant whose value depends on the temperature profile. l is the cantilever’s length and w its effective width. γ is the thermal expansion coefficient of a layer, t is its thickness, λ is the thermal conductivity, and their subscripts refer to the BMC layer. K can be expressed in terms of the layers’ thickness and Young’s Moduli $E_{i}$:

(4) $$K=4+6\left(\frac{t_{1}}{t_{2}}\right)+4{\left(\frac{t_{1}}{t_{2}}\right)}^{2}+\;\frac{E_{1}}{E_{2}}\;{\left(\frac{t_{1}}{t_{2}}\right)}^{3}\;+\frac{E_{2}}{E_{1}}\left(\frac{t_{2}}{t_{1}}\right),$$

Numerous works have developed BMC chip calorimeters, in which silicon nitride was a recurrent choice owing to its robustness and excellent mechanical, chemical, optical, electrical, and thermal properties. Silicon nitride was hence employed in combination with Al [176,177,178,179] or Au [180,181,182,183,184] as the cantilever’s bilayer and picowatt resolutions were reported [181,182]. Simulation and mathematical models have also been developed, dedicated to the measurement of a single biological cell’s heat capacitance [185].

To circumvent the complexity of measuring liquid samples without compromising the insulating advantages of measurements in vacuum, some of the developed devices were suspended microchannel cantilevers [186,187,188]. Within these BMC designs, a channel is embedded to confine the liquid samples. These devices have been used for photothermal identification of liquid reagents [178], or measuring the heat capacity of liquids [179].

BMCs are not the only design adopted for static mechanical chip calorimeters. A drum structure has been implemented to probe thermal transport [189]. This chip calorimeter employs a 20 nm-thick silicon nitride membrane and has a 1 mm^2^ sensing area, achieving a resolution of 100 pW.

### 4.2. Dynamic Mechanical Chip Calorimeters

As in the static case, various microresonator structures can be used as calorimeter sensors by detecting temperature-dependent changes in resonance. This approach holds great promise for chip calorimetry as the resonant frequency of a dynamic mechanical transducer can be detected with high precision [190]. To achieve such levels of precision, however, the devices must be operated in vacuum, ensuring minimal energy dissipation and high quality factors. Numerous devices targeted the measurement of biological samples; however, biological entities necessitate examination within their native fluidic environment. This requirement compels the operation of resonator devices within fluid media, which diminishes quality factors and sensor efficiency. Consequently, the design must incorporate microfluidic confinement systems to enable dynamic measurements of biological samples in vacuum conditions, thereby eliminating fluidic damping effects.

To address this challenge, one of the proposed designs is Si resonator beams integrated in a microfluidic chip for the thermal measurement of a single cell [191,192,193]. In this design, the beam resonators are enclosed in vacuum and connected to a microfluidic stage where the sample is flown. When in contact with the beam resonator, the heat generated by the captured cell is conducted to the sensor in vacuum, inducing a shift in its resonant frequency. The measurement can hence be performed, avoiding heat loss and vibration damping. The resonance frequency temperature responsivity of the cantilever devices—also referred to as the Temperature Coefficient of the Resonant Frequency (TCRF)—can be expressed as follows [193]:

(5) $$\frac{1}{f}\frac{\partial f(T)}{\partial T}=\frac{1}{2}\alpha_{E}-\alpha_{l}-\frac{1}{2}\alpha_{\rho },$$

where $\alpha_{E}$ and $\alpha_{\rho }$ are the temperature coefficients of Young’s modulus and the density, respectively, and $\alpha_{l}$ is the thermal expansion coefficient.

Alternatively, channels can be embedded within the resonator device—similar to those seen in static mechanical chip calorimeters. Suspended Microchannel Resonators (SMRs) can serve as calorimeters [194]. The relative frequency shift inversely depends on fluid flow rates because the device cools via convection. The resonance frequency temperature responsivity of the cantilever includes a correction factor accounting for fluid flow within the embedded channel, which predominantly influences its responsivity, thereby making this design a great candidate for calorimetric applications. This responsivity is expressed as [195]:

(6) $$\frac{1}{f}\frac{\partial f(T)}{\partial T}\approx \frac{1}{2}\alpha_{E}+\frac{1}{2}\alpha_{l}-\frac{1}{1+\frac{A_{S}\rho_{S}}{A_{F}\rho_{F}}}\left(\frac{3}{2}\alpha_{l}+\frac{1}{2}\frac{1}{\rho_{F}}\frac{\partial \rho_{F}\left(T\right)}{\partial T}\right),$$

where $\alpha_{E}$ and $\alpha_{l}$ are the temperature coefficients of Young’s modulus and the thermal expansion coefficient, respectively. A is the cross-sectional area and ρ is the density. Subscripts S and F refer to the solid constituting the channel and the fluid samples flowing inside the channels, respectively.

Other dynamic mechanical chip-calorimeters have targeted broader applications of heat measurements. In these devices, liquid samples are not involved, making vacuum measurements inherently feasible. Micro string resonators were established as promising candidates for high-resolution temperature detection. The eigen frequency $f_{0}$ responsivity at temperature $T_{0}$ is then given by [196]:

(7) $$\frac{1}{f_{0}}{\frac{\partial f_{0}}{\partial T}|}_{T=T_{0}}\approx -\frac{E(\alpha_{str}-\alpha_{sub})}{2\sigma_{0}},$$

where $\alpha_{str}$ and $\alpha_{sub}$ are the thermal expansion coefficients of the string and substrate, respectively, and E is Young’s modulus. L and $\sigma_{0}$ are the string’s length and tensile initial stress at $T_{0}$.

Various devices were also developed, namely, resonators for IR detection with a microplate [197,198], -drum [199] or -trampoline designs [200], -trampoline resonators for sub-terahertz detection [201], and self-oscillating cantilever calorimeters [202].

Microstring, -drum, and -trampoline resonators were also employed in photothermal microscopy and spectroscopy, where absorbed power is used as a transduction parameter for biochemical recognition of different compounds [203,204,205,206,207,208,209,210,211,212,213].

### 4.3. Design and Performance Considerations

Converting the mechanical resonator’s motion into a measurable signal and, conversely, enabling actuation of motion constitute the process of motion transduction. Various transduction techniques can be employed, either externally or fully integrated on chip. Some methods, such as capacitive, magnetomotive, optical, and piezoelectric transduction, enable both actuation and detection.

Optical transduction, for instance, offers high flexibility in the design and fabrication of the resonators. It, however, requires bulky instrumentation and precise alignment. It additionally dictates geometrical constraints and is fundamentally limited at the nanoscale by optical diffraction. In contrast, electrical transduction techniques are highly suitable for miniaturization but often introduce additional fabrication complexities [214]. Furthermore, when selecting a transduction approach for chip calorimetry, one must consider a method to avoid local heating in the sensor and to minimize heat loss in the system. Therefore, piezoelectric transduction represents a compelling choice as it provides a linear response, full on-chip integration, and minimal energy dissipation [215,216].

**Figure 8:**
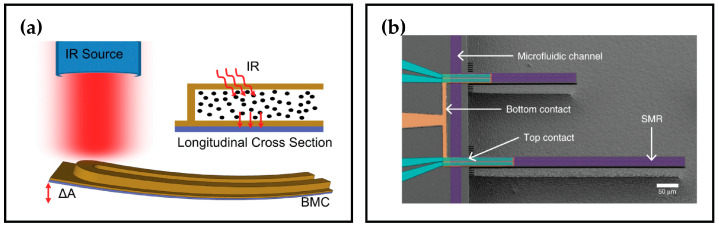
Suspended beam devices with embedded microchannels used in sensing applications in different modes: (**a**) Static: a BMC filled with a sample is irradiated with a tunable IR source, when the sample absorbs IR radiation at their resonance frequency, localized heat is generated due to nonradiative decay process causing the deflection of the gold (in blue)/SiN (in gold) BMC, identification of the liquid sample can then be done by [plotting the amplitude of the beam deflection as a function of IR wavenumber [178]; (**b**) Colored Scanning Electron Microscope (SEM) of an array of two SMRs with their piezoelectric electrodes on top. The microfluidic channel is in purple, the bottom electrode in orange and the top electrode in cyan [194].

Figure 8 illustrates suspended microchannel beam sensors employed for various applications. Meanwhile, Table 5 summarizes mechanical chip calorimeters reported in the literature, operated in either static or dynamic modes, along with their reported responsivities.

## 5. Challenges

As outlined in the preceding sections, chip calorimetry has undergone significant development, encompassing a broad range of sensing methodologies. Optical, electrical, and mechanical approaches each offer distinct advantages; however, they also present inherent limitations. In the context of single-cell analysis, these limitations become increasingly evident, as system design must adhere to rigorous biological and physical constraints. Consequently, the selection of heat-sensing modality is inherently dependent on the specific application, necessitating careful evaluation of performance trade-offs. We try to summarize the principal strengths and limitations of these sensing principles in the domain of single-cell calorimetry in Table 6.

Optical chip calorimeters offer excellent spatial resolution and enable contactless measurements, making them attractive candidates for various applications. However, their performance is limited by wavelength restrictions. In single-cell analysis, which often involves aqueous environments, optical methods encounter additional challenges from diffraction and scattering effects that compromise signal integrity. Furthermore, some implementations also require specialized probes, which increases experimental complexity and can introduce perturbations such as phototoxicity or unintended effects on cell behavior. Additionally, optical chip calorimeters are also quite diverse, addressing temperature differences rather than reporting system parameters to determine their power resolution, which complicates their benchmarking against other chip calorimeter technologies.

Electrical chip calorimeters are the most established devices within the field. When applied for biological measurements, resolutions of nW are achieved. Their use for single-cell measurements presents specific limitations. The choice of materials for resistance-based or thermocouple chip calorimeters must meet biocompatibility requirements, which can limit design flexibility. Additionally, the overall resolution is often constrained by the performance of the readout electronics, which can hinder the detection of the tiny thermal signals produced by individual cells.

Mechanical chip calorimeters hold significant potential for single-cell calorimetry. This potential is attributed to the inherent enhanced insulation provided by suspension—further elaborated in this section—as well as the capability to achieve highly accurate measurements of resonant frequency, thereby enabling high-resolution detection of signals from individual cells. In this context, nW resolutions can be achieved, offering a competing alternative to electrical chip calorimeters. For optimal functionality, piezoelectric transduction offers a rapid, linear, and non-dissipative approach that does not interfere with calorimetric measurements. However, the integration of fully on-chip piezoelectric transduction introduces additional complexities in the fabrication process.

Despite these advancements, numerous challenges persist across all chip calorimeter platforms. Device complexity introduces competing design constraints, which require trade-offs to be made. This section explores the primary challenges in chip calorimetry, reviews current strategies for their mitigation, and emphasizes the inherent compromises associated with each approach.

### 5.1. Insulation and Temperature Control

Insulation is a critical factor in calorimetry, as it minimizes heat loss to the surroundings and shields the sample from external disturbances that can distort the measurement. This aspect is particularly important for chip calorimeters, where the large surface-to-volume ratio increases susceptibility to thermal losses [24]. Efficient insulation, therefore, enhances sensitivity by preserving a larger detectable heat change for the same thermal event, thereby improving the signal. Moreover, insulation plays a key role in temperature control, since most chip calorimeters lack feedback regulation due to the technical challenges of integrating complex control systems into chip-scale devices. Consequently, significant improvements are still needed for chip calorimeters to reach the level of thermal control achieved by their bench-top counterparts [36].

In chip calorimeters, heat transfer occurs through four modes: conduction, evaporation, convection, and radiation, with the latter two being comparatively less significant. The heat transferred by radiation is proportional to the absolute temperature raised to the fourth power. Addressing exceedingly small temperature variations within the framework of chip calorimetry renders radiation’s contribution to heat loss negligible. Convection by air is circumvented by performing chip calorimetric measurements in vacuum. When liquid flow is involved in the chip calorimetric system, heat loss by convection poses a greater concern—an aspect examined in the next section, which discusses sample handling.

Complete elimination of heat transfer by conduction is unfeasible; however, reducing its effect can be achieved through various strategies. One approach would be through the architecture of the sensing area, achieving insulation by suspension. Numerous optical [65,66] and electrical chip calorimeters adopted the suspended sensing area design (Table 2 and Table 4), while being an innate feature for mechanical chip-calorimeters. Excessively reducing one or two dimensions of the sensing area by using one- or two-dimensional structures can help with better insulation and increased sensitivity; however, it will be unfeasible integrate liquid samples in the case of biosensing [36].

To mitigate evaporation effects, closed-chamber calorimeters are usually preferred over open-chamber designs. In this configuration, a microfluidic system confines the liquid sample within a channel, either for delivery into a reaction chamber or for direct measurement within the channel. Closed-chamber systems additionally enable perfect encapsulation in vacuum environment [34], thereby improving insulation. While this approach addresses evaporation-related issues, closed-chamber calorimeters present their own challenges. Beyond the complexities introduced by the microfluidic system—discussed separately in upcoming subsections—the microfluidic architecture increases thermal conductance to the surroundings, has a larger heat capacity than the open-chamber configuration [35], and adds mass to the calorimeter sensor [36]. These aspects are of considerable significance as they pertain to the system’s responsivity. The specific heat and the mass of the sensing system determine its dynamic responsivity, whereas its thermal conductivity (or, inversely, thermal resistivity) defines its steady-state responsivity.

To address these challenges, one approach would be to use low thermal conductivity materials such as Pyrex [117], or polymers like polydimethylsiloxane (PDMS) [74,116,120,217], polymethylmethacrylate (PMMA) [136], parylene [29,87,91,126], SU-8 [218], and polyimide [97]. Nevertheless, porous polymers are unsuitable for vacuum measurements. Si-based materials can also be used, despite having relatively higher thermal conductivity. Insulation can be improved by decreasing thickness and conducting measurements in vacuum. Si-based materials additionally exhibit low specific heat values and are compatible with various microfabrication processes [24]. Furthermore, complex thin 3D wall structures can also be employed to isolate the system with minimal added mass [95]. Table 7 reports on the thermal properties of the commonly used materials in chip calorimetric systems.

In summary, insulation and temperature control are key challenges in chip calorimetry due to the system’s high susceptibility to thermal losses and limited active temperature regulation. While radiation is negligible due to small temperature changes, convection is avoided by vacuum operation and flow control. Conductive heat loss remains primary, mitigated by structural design like suspended membranes and scaling. Closed chambers reduce evaporation and enable environmental insulation, but increase thermal conduction, heat capacity, and mass, affecting responsivity. Material choice is complex, balancing low conductivity, fabrication compatibility, and vacuum suitability. Overall, effective insulation without sacrificing sensitivity and dynamic performance remains a key limitation preventing chip calorimeters from matching the thermal control of macrosystems.

### 5.2. Sample Preparation and Sample Handling

Sample preparation is a crucial step in chip calorimetry, as it is recognized as a significant source of error in bioanalytical processes [220]. While appropriate collection and storage methods are essential, pre-measurement treatment is often complex and requires considerable time and resources. The diversity and variability of biological samples complicate the establishment of universal and standardized preparation protocols [221,222]. Challenges frequently arise from the presence of target analytes at exceedingly low concentrations in the primary extracted sample, thereby necessitating preconcentration. The complex matrices of biological samples, including blood, urine, saliva, and plasma, in which numerous compounds may influence sample stability, introduce further challenges. Additional sample clean-up is often required to prevent degradation from endogenous factors such as enzymatic activity and cell death [220].

A central challenge in advancing single-cell calorimetry lies in integrating measurements within the broader context of intracellular communication. Studies have demonstrated that signals exchanged between cells are often transient, low in abundance, and highly sensitive to diffusion, necessitating confined microfluidic environments for accurate detection [30]. This highlights a major limitation: isolating a single cell for calorimetry may disrupt or obscure the signaling processes that regulate metabolic activity. Therefore, achieving true single-cell resolution is not solely a matter of improving resolution, but also about preserving or reconstructing relevant interaction networks. Approaches should combine calorimetry sensing with microfluidic platforms capable of capturing biochemical signaling and cell interactions, enabling a more comprehensive and context-aware interpretation of single-cell activity.

Sample handling refers to the methods of transferring samples into the measurement space. As briefly discussed in the previous subsection, chip calorimeters can be implemented in two configurations: open- and closed-chamber chip calorimeters. In both designs, mixing is a vital aspect to consider. Mixing can be achieved in one of two approaches. Active mixing is accomplished by supplying energy from external sources [84,85,223,224,225,226]. Passive mixing is implemented in closed-chamber calorimeters via geometric features engineered for this purpose [227,228]. While the available mixing approaches are diverse, the selection is limited by a few requirements. First, the mixing time should be shorter than the thermal dissipation time constant. Additionally, mixing should not interfere with the measurement by altering the biochemistry, affecting the droplets’ reactions, or introducing thermal noise to the system [35].

In open-chamber designs, samples are delivered via micropipettes or inkjets, producing droplets ranging from a few µL to hundreds of pL [24]. Micropipettes are frequently used [50,70,122,123,139] but are relatively slow and incapable of handling multiple samples simultaneously, contrary to inkjets [25]. The higher speed of inkjets, however, comes at the cost of introducing additional heat into the system, thereby interfering with the measurement [35]. Open-chamber calorimeters also face the added challenge of sample loss through evaporation. One way to circumvent this challenge is to implement a “Virtual Reaction Chamber (VRC)” where the sample droplets are covered with oil to prevent evaporation [101,107].

Closed-chamber chip calorimeters address the challenges associated with evaporation while also providing greater control over the sample volumes using pumps, valves, and channels [24]. Numerous microfluidic designs have been developed not only for chip calorimeters but also for chip biosensors and LOC applications, employing various materials and fabrication methods [229,230,231,232,233]. For chip calorimetry, however, additional aspects must be considered, such as material choices for optimal insulation and reduced device mass—as discussed in the previous subsection. Another key aspect is the specific functionalities needed for the intended measurement application. For example, some applications may require on-chip cell culture, a feature that can also be achieved with microfluidic chips [37,234].

Within closed-chamber calorimeters, a sub-classification can be made between flow-through and stationary devices based on the motion of the liquid during calorimetric sensing. In flow-through designs, measurements are performed as the liquid sample passes through the detection area, whereas in stationary designs, the measurement is conducted on a confined, non-flowing sample volume within the sensing area [36]. Stationary chip calorimeters are reported to have a higher resolution [37], as liquid flow highly contributes to heat dissipation by convection [194]. Nonetheless, the choice of the system will depend on the target application, as stationary designs are not well-suited for fast, high-throughput measurements.

For chip calorimetry at the single-cell level, cell capture is a crucial functionality to incorporate. In closed-chamber systems, manipulation can be achieved dielectrophoretically [235,236,237], magnetically [238,239,240], acoustically [241,242,243], hydrodynamically [244,245,246,247,248,249] or through droplet technology [250,251,252,253]. However, these methods may interfere with calorimetric measurements, reducing resolution and/or increasing the time constant. One proposed approach is to separate the capture and the detection areas [37]. This decoupling will preserve the integrity of the calorimetric measurement from any external perturbations.

Closed-chamber designs, despite offering numerous advantages, entail greater fabrication and operational complexity. Fabrication challenges arise from the additional process steps required to implement the on-chip microfluidic system, and to integrate a macroscale fluidic interface, while operational difficulties stem from the use of pumps, syringes, mixers, and valves. Additionally, the dead sample volume required for the operation of the microfluidic system results in the consumption of more sample than is needed for the measurement. A possible solution is to employ capillary microfluidic systems that enable full on-chip integration, simplify operation, and help reduce the required sample volume [254].

To summarize, sample preparation and handling remain critical challenges in chip calorimetry, especially for biological samples with low analyte concentrations, complex matrices, and intercellular signaling that regulate their activity. The absence of standardized protocols and the need for pre-treatment increase time, cost, and potential errors. Open- and closed-chamber designs involve trade-offs between simplicity and insulation. While closed-chamber systems improve sample control and reduce evaporation, they introduce higher thermal losses, fabrication complexity, and greater sample consumption. Additional constraints arise from mixing requirements, which must be rapid yet non-disruptive to measurements, all while accounting for cell–cell interactions. Application-specific needs, such as cell culture, further complicate system design. Overall, ensuring accurate, low-noise sample handling without compromising sensitivity or throughput remains a significant limitation.

### 5.3. Measurement Repeatability, Reproducibility, and Resolution

The intricacy of chip calorimeter systems presents multiple challenges for data interpretation and measurement reliability. While several of these issues have been thoroughly discussed in the previous subsections, the challenges of the measurement repeatability, reproducibility, and resolution remain to be addressed.

Repeatability and reproducibility are two related yet distinct aspects, essential for ensuring the quality of a measurement system [255]. Repeatability refers to the consistency of results from a measurement procedure under the same conditions within a single laboratory or by the same operator, whereas reproducibility refers to the consistency of the results from a measurement procedure under different conditions, such as different equipment, laboratories, or operators [256].

Sample handling can significantly impact both repeatability and reproducibility. For example, variations between measurements may arise from sample mixing, where incomplete mixing or frictional heating during the process can interfere with the recorded heat values [35,255]. Additionally, since chip calorimeters operate with µL–pL volumes, minor variations in sample loading or preparation can lead to substantial inconsistencies in measurements [36,220,221,222]. Finally, the analyte’s thermal mass is an important factor to consider. Because aqueous solutions have high heat capacities, small variations in the solution can lead to significant variation to the heat loss with sample volume and thus affect the measured temperature change [36].

The intricate fabrication of chip calorimeters is another significant source of reproducibility issues. The multistep processes often involve several lithography, etching, deposition, and—at times—doping steps. The process is hence not only time-consuming and costly, but also prone to batch-to-batch variations. Furthermore, discrepancies between research groups and studies result from differences in calibration, environmental conditions, and protocol complexities.

The demand for high-performance calorimeters capable of resolving nano- and pico-watt heat power changes, enabling heat measurement at the single-entity level, has been, and remains, a major driving force for the development of chip calorimeters. However, despite the continuous pursuit of higher resolution, most of the reported values remain theoretical estimates that cannot be guaranteed in real applications, where thermal signals from various environmental or operational factors may introduce disturbances that can readily hinder resolution [36].

Aqueous environments additionally challenge the measurement resolution. The pico- and sub-picowatt resolutions reported in the literature were achieved using one- or two-dimensional suspension systems [99,105,181,182,189]—a design not compatible with aqueous media measurements. Therefore, guiding heat from the measured sample within a channel into a suspended beam [191,192,193], or embedding microfluidic channels into suspended beams [178,179,194,195], offers an alternative to optimize insulation for liquid samples through suspension and vacuum measurements, and enables single-cell measurements [191]. Further improvement in resolution can be achieved through careful engineering of these designs’ dimensions.

In summary, measurement repeatability, reproducibility, and resolution in chip calorimetry are restricted by both operational and fabrication-related factors. Variations in sample handling, such as mixing efficiency, loading accuracy, and thermal mass effects, can greatly influence measurement consistency, especially at nano- and pico-scale volumes. Fabrication complexities also cause batch-to-batch variability and interlaboratory differences due to variations in process, calibration, and experimental conditions. Although high theoretical resolutions have been reported, actual performance is often reduced by environmental and system noise. Furthermore, achieving high resolution in aqueous environments remains challenging due to design constraints and increased thermal losses. Overall, ensuring reliable, high-resolution measurements under realistic conditions continues to be a significant challenge in chip calorimetry.

### 5.4. Fabrication, Throughput, and Commercial Use

The fabrication of chip calorimetry systems is a complex and costly process, which challenges their large-scale implementation and commercial availability. While fabrication is a critical factor, additional limitations encompass reliability, ease of operation, versatility, and flexibility for automation [36]. The question of throughput also persists; although chip calorimeters have been previously employed for high-throughput drug screening [77,84], significant progress is still required to achieve true high-throughput, particularly at the single-entity level.

## 6. Conclusions and Outlook

Chip calorimetry is a powerful approach for precise, real-time thermal analysis at the micro and nanoscale. As elucidated in this manuscript, chip calorimetry has the potential to significantly enhance our understanding of biological processes and to contribute to advancements in disease diagnosis. When targeting analysis at the single-cell level, the true value lies not only in improving the measurement resolution, but in enabling― through microfluidic integration―a more integrated understanding of biological systems, where thermal signals can be interpreted alongside biochemical and intracellular communication processes. In this context, chip calorimetry emerges as a key enabling technology for bridging physical measurements with functional biological insight.

In this paper, we demonstrated that recent years have witnessed considerable progress across various operational principles of chip calorimetry. Innovations in heat sensing mechanisms, microfluidic integration, and noise reduction strategies have collectively elevated device performance to a high standard. However, each heat-sensing approach retains intrinsic limitations: optical calorimeters, while offering high spatial resolution and non-contact operation, remain constrained by wavelength operating range and signal degradation in aqueous media. Electrical calorimeters, though well established, face biocompatibility constraints and resolution limits imposed by readout electronics. Mechanical calorimeters, despite their high resolution and advantageous thermal insulation, introduce additional fabrication and integration complexities, particularly when incorporating piezoelectric transduction.

Despite this progress, several challenges still exist. The main ones include: (i) achieving effective thermal insulation and temperature control, (ii) ensuring consistent sample preparation and handling, and (iii) improving repeatability, reproducibility, and resolution—all of which remain key technical hurdles. Aqueous environments exacerbate these constraints further. The highest nano- and sub-nanowatt resolutions are typically achieved with suspended designs, which are not directly compatible with liquid measurements. Guiding heat from a microfluidic channel into a suspended structure or embedding channels within the suspended sensing area can help circumvent this challenge. In these designs, resolution strongly depends on careful geometric optimization. Additionally, to enable high-throughput capabilities and support robust commercial deployment, chip calorimeters require significant improvements in system integration and manufacturability.

Looking forward, multiple development routes hold promise for addressing these open points. Advancing measurement systems to enhance sensitivity and resolution remains a priority, alongside improving microfabrication techniques through scalable and cost-effective processes. Approaches such as low-cost 3D printing for microfluidic fabrication offer attractive opportunities for accessible, LOC platforms. Equally important is full on-chip integration of sensing, microfluidics, and encapsulation, which will be critical for industrial adoption.

For the next generation of chip calorimeters, automation will play a key role, as partly demonstrated by some studies using automated microfluidic control. This will significantly improve repeatability and help reduce operational errors. Measurement and data analysis and automation, combined with calorimetric array architectures, will enable high-throughput screening and the acquisition of large datasets, which will lessen the effects of sample variability. Notably, there is a trend toward going beyond calorimetry alone to develop multi-parametric sensor platforms that can combine parameters, such as mass/density and thermal properties, offering comprehensive, multiparametric characterization of biological samples within a single chip.

Overall, while several challenges remain, chip calorimetry continues to show strong potential for biological and biomedical applications. Chip calorimetry proves flexibility to evolve from a standalone measurement technique toward an integrated, context-aware analytical platform. This evolution is particularly critical for single-entity analyses, which is a goal that is not far from being broadly available. For the next few years, we expect a consistent push towards innovation in device architecture, automation, and scalable fabrication. All these improvements will, in the end, translate chip-calorimetry from research settings toward broader commercial and clinical use.

## Figures and Tables

**Table 1:** The values of the heat power of single living cells/organisms reported in the literature.

Living Cell	Heat Power	Reference
Human lymphocytes	5 pW	[27]
3T3 mouse fibroblasts	17 pW	
HeLa-53G	31 pW	
Rat white adipocytes	40 pW	
Human melanoma, H1477	80 pW	
Rat hepatocytes	102 pW	[28]
Kidney cell	0.3 nW	[29]
Neuron cell	0.3 nW	
Amoeba	1.3 nW	
Stimulated muscle cell	2.3 nW	
Stimulated myocardial cell	2.8 nW	
Stimulated brown fat cell	3 nW	
Tetrahymena	3.1 nW	

**Table 2:** Summary of the Resistance-based chip calorimeters.

References	Year	Thermometry		TCR (K^−1^)	Insulation by Suspension	Liquid Samples	Temperature Resolution	Power Resolution
		Type	Materials					
[82]	1995	Thermistor	Doped Polysilicon	−1.7%	No	Yes	5000 μK *	∼200 μW *
[84] ◊	2004		n^+^ Amorphous Silicon	2.8%	Yes	Yes	500 µK	∼2 μW *
[85,86] ◊	2008–2009		Vox	2.7%	Yes	Yes	60 µK	60 nW
[83]	2011		B^+^ Polysilicon	0.07%	Yes	Yes	∼200 μK *	5 nW *
[77,79,89,90,93]	2016–2022		Vox	−(2.5–2.8)%	Yes	Yes	60 µK	40 nW
[87]	2016		Vox	−2.2%	Yes	Yes	50 µK	570 pW
[88]	2017		Vox	−1.3%	Yes	Yes	1000 μK	75 nW *
[91]	2020		Vox	−3.4%	Yes	Yes	400 µK	∼5 nW *
[105] ¤	2014	RTDs	NbN_x_	0.67%	Yes	No	∼250 μK *	∼1.5 pW *
[96]	2014		Ni	0.11%	No	Yes	25,000 μK □	5 μW □
[100,101]	2014–2020		Al	0.315%	Yes	Yes	150 µK	40 nW
[106]	2016	PN Junction	n SOI wafer and B^+^ Si	1.4%	Yes	Yes	1100 μK	75 nW

* Values marked with an asterisk are calculated from reported data under the assumption that the measurements are limited by the Johnson noise. This assumption is not strictly valid at low frequencies, where flicker noise ($\frac{1}{f}$) dominates over thermal noise; however, it remains adequate for the purpose of relative benchmarking in this table. ∼ Denotes an approximate value using an assumed signal-to-noise ratio (SNR) of 5:1 or conductance values from literature for similar materials and thicknesses. ¤ Not intended for biological applications. ◊ Refers to the same device but with a different thermistor material. □ Device limited by source/meter.

**Table 3:** Seebeck coefficients of commonly used materials (relative to Pt −5 µV/K) [110,111,112].

Materials	Seebeck Coefficient (µV/K)
Si	440
Cr	21.8
Ni	−15
Al	3.5
W	7.5
Pb	4
Cu	6.5
Bi	−72
Ti	1
Au	6.5
Pt	0
Pd	11
Constantan	−35
Nichrome	25

**Table 4:** Summary of the thermocouple-based chip-calorimeters reported in the literature.

Reference	Year	Materials	Insulation by Suspension	Liquid Samples	Responsivity (V/W)	Power Resolution
[127]	1993	Al/P-type Silicon	Yes	Yes	1	0.2–0.4 µW
[128]	1994	Al/Si	Yes	Yes	8	∼25 nW *
[129,131]	2000–2007	Al/p^+^ Polysilicon	Yes	Yes	7	∼25 nW *
[123,124,125]	2002–2004	Au/Ni	Yes	Yes	2	10–25 nW
[117]	2004	Au/B^+^ Polysilicon	Yes	Yes	1	∼250 nW *
[5,135,136,137,138,140,141,143,145,146,147,149,150,151,152]	2005	BiSb/Sb	Yes	Yes	8	50 nW
[73,116]	2008	Cr/Ni	Yes	Yes	1	∼360 nW *
[132]	2009	Al/Polysilicon	Yes	No	2430	3.4 µW
[126]	2009	Au/Ni	Yes	Yes	7	4 nW
[113]	2009	p Polysilicon/n Polysilicon	Yes	Yes	11	200 nW
[158]	2011	p Polysilicon/n Polysilicon	Yes	Yes	5	∼500 nW *
[71,74,144]	2012–2015	Bi/Sb	Yes	Yes	8	∼10 nW *
[148]	2015	Bi/Sb	No	Yes	5	20 nW
[121]	2017	Bi/Ti	Yes	Yes	7	∼10 nW *
[122]	2019	Bi/Ti	Yes	Yes	45	∼1 nW *
[8]	2019	Bi/Sb	No	Yes	5	∼20 nW *
[154]	2020	Bi/Sb	Yes	Yes	5	50 nW
[167]	2025	Bi_2_Te_3_	No	Yes	1	5 μW

* Values marked with an asterisk are calculated from reported data under the assumption that the measurements are limited by the Johnson noise. This assumption is not strictly valid at low frequencies, where flicker noise ($\frac{1}{f}$) dominates over thermal noise; however, it remains adequate for the purpose of relative benchmarking in this table. ∼ Denotes an approximate value using an assumed signal-to-noise ratio (SNR) of 5:1 and/or noise floors derived from literature with comparable materials. Values are rounded.

**Table 5:** Summary of the mechanical chip calorimeters reported in the literature.

Reference	Year	Type	Liquid Samples	Responsivity	Resolution
[176,177]	1994	Static	No	0.18 Å/nW	100 pW
[180,183,184]	2010–2016		No	0.09 Å/nW *	∼200 pW *
[181] ¤	2011		No	2.5 Å/nW *	4 pW
[189] ¤	2024		No	35 Å/nW *	100 pW
[191,192,193]	2012–2016	Dynamic	Yes	−77 ppm/nW *	70–80 nW *

* Values marked with an asterisk are calculated from reported data. ∼ Denotes an approximate value based on noise-floor values reported in the literature for similar systems. ¤ Not intended for biological applications. Values are rounded.

**Table 6:** A summary of the advantages and limitations of each of the three heat-sensing principles for single-cell chip calorimetry.

	Optical Chip Calorimeters	Electrical Chip Calorimeters	Mechanical Chip Calorimeters
Advantages	Excellent spatial resolution Non-contact measurement	The most established technology Resolution of a few nW	Enhanced insulation by inherent suspension Precision in frequency detection Resolution of a few nW
Limitations	Diffraction and scattering Probes and phototoxicity Difficult to benchmark	Restriction in materials for biocompatibility Resolution limited by electronics	Integrated piezoelectric transduction complicates fabrication

**Table 7:** Thermal properties of commonly used materials in chip calorimeters. [24,35,219].

Materials	Thermal Conductivity (W/m·K)	Specific Heat (J/g·K)
Air	0.026	1.01
PDMS	0.15	1.46
PMMA	0.19–0.24	1.46–1.47
Parylene	0.082	0.71
SU-8	0.20	1.50
Polyimide	0.12	1.09
Si	130	0.71
Si_3_N_4_	15–30	0.70
SiO_2_	1.1–1.4	1.0

## Data Availability

The original contributions presented in this study are included in the article. Further inquiries can be directed to the corresponding author.

## Abbreviations

The following abbreviations are used in this manuscript:

ITC	Isothermal Titration Calorimetry
DSC	Differential Scanning Calorimetry
TMDSC	Temperature Modulated Differential Scanning Calorimetry
µTAS	Micro Total Analysis Systems
IR	Infra-red
PCR	Polymerase Chain Reaction
LC	Liquid Crystal
ESR	Electron Spin Resonance
ODMR	Optically Detected Magnetic Resonance
QD	Quantum Dot
NV	Nitrogen Vacancy
OWiC	Optical Wireless-Integrated Circuit
PV	Photovoltaic
LED	Light Emitting Diode
LSPR	Localized Surface Plasmon Resonance
LFA	Lateral Flow Assay
NP	Nanoparticles
LOC	Lab-on-chip
RTD	Resistive Temperature Detector
TCR	Temperature Coefficient of Resistance
TFTC	Thin Film Thermocouple
SNR	Signal-to-Noise Ratio
BMC	Bimaterial Cantilever
TCRF	Temperature Coefficient of Resonant Frequency
SMR	Suspended Microchannel Resonator
SEM	Scanning Electron Microscope
PDMS	polydimethylsiloxane
PMMA	polymethylmethacrylate
VRC	Virtual Reaction Chamber
